# Current Status of Trace Metal Pollution in Soils Affected by Industrial Activities

**DOI:** 10.1100/2012/916705

**Published:** 2012-05-03

**Authors:** Ehsanul Kabir, Sharmila Ray, Ki-Hyun Kim, Hye-On Yoon, Eui-Chan Jeon, Yoon Shin Kim, Yong-Sung Cho, Seong-Taek Yun, Richard J. C. Brown

**Affiliations:** ^1^Department of Environment and Energy, Sejong University, Seoul 143-747, Republic of Korea; ^2^Department of Farm Power and Machinery, Bangladesh Agricultural University, Mymensingh, Bangladesh; ^3^Korea Basic Science Institute, Seoul Center, Anamdong, Seoul 136-713, Republic of Korea; ^4^Institute of Environmental and Industrial Medicine, Hanyang University, Seoul 133-791, Republic of Korea; ^5^Department of Earth and Environmental Sciences, Korea University, Seoul 136-701, Republic of Korea; ^6^Analytical Science Division, National Physical Laboratory, Hampton Road, Teddington, TW11 0LW, UK

## Abstract

There is a growing public concern over the potential accumulation of heavy metals in soil, owing to rapid industrial development. In an effort to describe the status of the pollutions of soil by industrial activities, relevant data sets reported by many studies were surveyed and reviewed. The results of our analysis indicate that soils were polluted most significantly by metals such as lead, zinc, copper, and cadmium. If the dominant species are evaluated by the highest mean concentration observed for different industry types, the results were grouped into Pb, Zn, Ni, Cu, Fe, and As in smelting and metal production industries, Mn and Cd in the textile industry, and Cr in the leather industry. In most cases, metal levels in the studied areas were found to exceed the common regulation guideline levels enforced by many countries. The geoaccumulation index (*I*_geo_), calculated to estimate the enrichment of metal concentrations in soil, showed that the level of metal pollution in most surveyed areas is significant, especially for Pb and Cd. It is thus important to keep systematic and continuous monitoring of heavy metals and their derivatives to manage and suppress such pollution.

## 1. Introduction

Industrial pollution has been and continues to be a major cause of environmental degradation. Numerous studies have already demonstrated that areas in close proximity to industrial activities are marked by noticeable contamination of air, soil, and water [1–3]. Hence, such activities can affect the air we breathe, the water we use, and the soil we stand on and can ultimately lead to illness and/or harm to the residents in the affected area.

Among various toxic substances released by industrial activities, heavy metals have been seen as a key marker because they may be analysed effectively and consistently in most environmental matrices. Unlike organic pollutants which may degrade to less harmful components as a result of biological or chemical processes, metals are not degradable by natural processes especially when elemental metallic content is considered [4]. The effects of metal pollution on local environments and organisms may therefore be substantial and long lasting in spite of extensive remediation efforts [4]. In fact, lead, cadmium, copper, manganese, and so forth have been commonly chosen as representative metals for which their concentrations in the environment may be used as reliable indices of environmental pollution [5].

In most parts of the world, large quantities of trace metals are directly discharged to nearby land and into surface waters. This activity adversely affects the quality of air, soil, and ground water, such that it becomes a subject of serious concern worldwide [6–8]. In recent years, many governments and policy makers have continued to strive for a more comprehensive understanding of environmental health hazards due to intensive industrial activities in order to inform future policy and abatement legislation [9]. In this paper, we intend to provide the results of a review of a survey of environmental pollution caused by industrial activities. Through an in-depth analysis of basic methodologies and relevant databases, we provide some insights into the fundamental aspects of metal pollution associated with industrial activities with a major emphasis on the soil matrix.

## 2. Status of Data Availability of Metal Pollution between Different Criteria: Matrix Types (Air, Soil, and Water) and Industry Types

**Figure 1:**
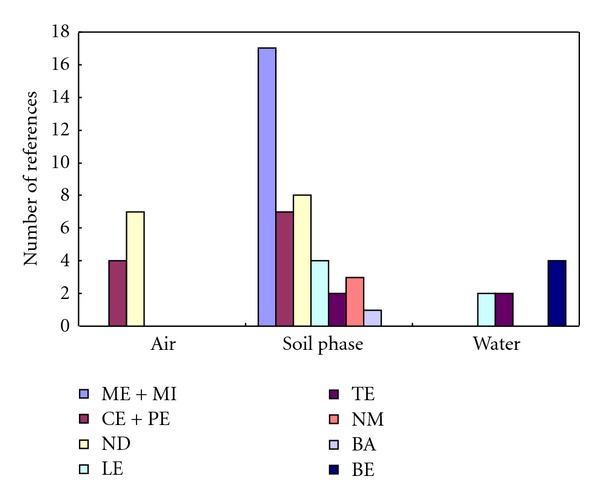
Source frequency of soil metal pollution data across different environmental matrices and with respect to different industry types (*n* = 61 references for comparison: refer to Table 1 for acronyms).

During the past few decades, industrial activities have increased greatly around the world with rapid economic growth. This has been accompanied by severe environmental pollution. Many studies have been carried out to evaluate the status of industrial pollution and its environmental impacts in a broad and aggregative manner. There are, however, very few studies that describe the impact of such pollution with respect to the spread of key pollutants across various environmental media. In order to build a database to assess the basic features of heavy metal pollution due to industrial activities, we conducted a literature survey of major articles dealing with this topic that have been published since 1996 (a total of 61 references). If these metal pollution data are sorted by matrix type, 42 of the articles dealt with the soil phase. The remaining ones dealt with the air (11) and water (8) phases. As the classification of the data sets surveyed is important, we used criteria provided by the International Standard Industrial Classification (ISIC) division of the United Nations (Table 1) [10]. The literature reviewed was chosen so as to provide a representative sample across industries, geographic area, and measured concentrations. It covered a range of industries including smelters, mining and metal (ME + MI), chemical and petrochemical (CE + PE), tannery (LE), ceramic and cement (NM), textile (TA), and industrial complexes (containing multiple generic process) (IC) of the studies in each category numbering 17, 7, 4, 3, 2, and 8, respectively. In case of air (*n* = 11), four case studies were mainly conducted near chemical and petrochemical (CE + PE) types, while all others concerned various industrial complexes (*n* = 7). Metal pollution in the water phase was investigated mainly in the brewery (BE = 4), tannery (LE = 2), and textile industries (TE = 2 cases).  Figure 1 shows the frequency of data availability for this survey as a function of industry type and between the different media.

## 3. Comparison of Experimental Approaches for Data Acquisition

### 3.1. Sampling

In this study, considering the availability of data, our analysis concentrated on the soil matrix. In this respect, we analyzed the basic methodological approaches employed for data acquisition in all the selected references. Based on this analysis, we evaluated the fundamental features of metal pollution in soil layers resulting from industrial activities. The basic information concerning the methodologies for sampling, sample treatment, and analysis for metal content is summarized in Table 2. In order to investigate the metal load onto soil, the distance between the sampling sites and the emission sources is a critical parameter. In general, there is a progressive decrease of metal concentrations with increasing distance from the source. Most of the studies reported soil sampling within a radius of 50 m to 2 km from industrial sources [15, 23, 30]. The collection of soil samples has commonly been made by random sampling [11] or grid sampling [14]. As seen in Table 2, grid sampling based on 1 km × 1 km squares has been adopted most commonly in many of these studies [12, 16, 22]. There are, however, some exceptions like the use of 20 m × 20 m grids (e.g. [28]). Most of the studies considered employing vertical sampling in the range of 0–5 cm [11] to 0–20 cm [33]. Soil samples were typically collected with a stainless steel spatula or auger and kept in PVC packages until analysis.

The metal data in soils derived from random and grid sampling are often used to represent an entire area. In this respect, grid sampling can be particularly useful when prior knowledge of the likely spatial variability is limited. This technique also avoids any sampling bias that could result from the collection of an unrepresentative average sample as a result high portion of subsamples from the same region. Two subtypes of grid sampling such as gridcell and gridpoint have commonly been employed (e.g., [34]).

### 3.2. Sample Preparation

Soil samples were commonly dried at room temperature and sorted via sieving (e.g., using a 2 mm sieve). They were then mineralized in a single acid like HNO_3_ within Teflon bombs in a microwave digester [12]. In many studies, however, authors preferred to use mixtures of various acids like (1) HCl, HNO_3_, and H_2_O_2_ ([11], (2) HCl, HNO_3_, and HF [14], (3) HCl, HNO_3_, HF, and HClO_4_ [16], (4) HCl, HF, and HClO_4_ [22], and (5) aqua regia [33]). Reagent blanks and standard reference soil samples were also analyzed to reduce experimental biases and properly validate each extraction method [33]. The digestion procedure used will depend on the species in the soil requiring digestion and the final analytical step. For instance, chromates may require more extreme digestion conditions, such as HF, to properly dissolve the chromium present. However, analysis techniques such as ICP-MS prefer final solutions with relatively low ionic contents and so HF (subsequently neutralized with HBO_3_) may cause instrumental drift during analysis [13].

### 3.3. Instrumental Detection

Flame/atomic absorption spectrometer (FAAS/AAS) is the dominant technique employed for metal analysis in soil [19, 22, 23, 27, 32]. In FAAS, either an air/acetylene or a nitrous oxide/acetylene flame is used to evaporate the solvent and dissociate the sample into its component atoms. The atoms of interest absorb light from a hollow cathode lamp (selected for the target element) as it passes through the cloud of atoms produced by the atomization process. The amount of absorbed light is measured and used to calculate the concentration of each metal of interest. Compounds of the alkali and transition metals can all be atomized with good efficiency yielding typical FAAS detection limits in the sub-ppm range.

AAS is also used in combination with a graphite furnace (GF) mode—known as GF-AAS [12, 25]. It is essentially the same as flame AA, except the flame is replaced by a small, electrically heated graphite tube, or cuvette, which is heated up to 3000°C to generate the cloud of atomized sample. The higher atom density and longer residence time in the tube yield improved detection limits (DLs) for GF-AAS in the sub-ppb range, which is by 3 orders of magnitude superior to flame AAS. However, because of the temperature limitation and the use of graphite cuvettes, the analytical performance for refractory elements is still somewhat limited. The techniques also exhibit a lower throughput than many of the more recent mass spectrometry (MS) techniques; this is because it is only able to determine one element at a time, unlike MS methods which can determine a range of elements at once.

Many authors have also used inductively coupled plasma with mass spectrometry (ICP-MS) or atomic emission spectrometry (ICP-AES) for the simultaneous analysis of multiple metals [14, 16, 20, 33]. For ICP/ICP-AES analysis, the system uses temperatures as high as 10,000°C to atomize even the most refractory elements with high efficiency. As a result, DLs for these systems can be orders of magnitude lower (typically at the 1–10 parts-per-billion level) than FAAS techniques. The ICP method can simultaneously screen for up to 60 elements in a single sample run of less than one minute, without any degradation of precision or detection limits. If run in a “sequential mode” ICPs can provide analytical results for about five elements per minute [35].

ICP-MS is a multielement technique that also uses an ICP plasma source to dissociate the sample into its constituent atoms or ions [35]. However, in this case, detection focuses on the ions themselves rather than the light that they emit. The ions are extracted from the plasma and passed into the mass spectrometer, where they are separated based on their atomic mass-to-charge ratio by a quadrupole or magnetic sector analyzer. In terms of DLs, ICP-MS can produce the best results (typically 1–10 ppt), followed by GF AAS (usually in the sub-ppb range), ICP-AES (of the order of 1–10 ppb), and FAAS (in the sub-ppm range). Being a mass spectrometric technique, ICP-MS also enables quantification by isotope dilution strategies for poly-isotopic elements, which can produce highly accurate results. However, mass spectrometric techniques may also suffer from isobaric mono- and polyatomic interferences which unless properly corrected can bias results [36].

Nondestructive methods for analysis such as X-ray fluorescence (XRF) and instrumental neutron activation analysis (INAA) have also been used commonly in many recent studies [16, 17, 24, 28]. In INAA, the sample is bombarded with neutrons, causing the elements to form radioactive isotopes. As the radioactive emissions and radioactive decay paths for each element are well known, one can determine the concentrations of the elements based on the information of spectral emission. This type of application is highly advantageous in that it does not destroy the sample and is generally matrixindependent, although often very difficult to calibrate accurately.

### 3.4. Quality Assurance (QA)

QA procedures are made to ensure that the approach is properly validated, under control at all stages, and employed appropriately. Validation of analytical methodologies via the measurement of matrix-certified reference materials is good examples of a necessary QA activity. Another important performance criteria in QA/QC is the method detection limit (MDL). The MDL is usually defined as the lowest quantity or concentration of a component that can be reliably detected for a given analytical method [55].

Standard reference materials (SRMs—generally NIST in the USA) or certified reference materials (CRMs—the term used by most other National Measurement Institutes (NMIs)) are materials used to check the quality and traceability of results and can be divided into two categories: calibrants and matrix RMs (as mentioned above). The former is mono- or multielemental standard solutions used for calibrating instruments and ensuring traceability in measurement results, while the latter is a material of a similar matrix to the sample being analyzed which has been certified for homogeneity and its content of relevant analytes (e.g., lead in dust). Matrix RMs are used for method validation, rather than for calibration.

Most of the reference materials used in soils have been formulated by several NMIs like National Institute of Standards and Technology (NIST), USA, Institute for Reference Materials and Measurements (IRMM, Belgium), and so forth. In the industrial soils of Baoji city, China, soil standards NIST-2709, 2710, and 2711 were used along with Canadian certified reference materials such as SO-1, 2, 3, and 4 [28]. In the industrial regions of Kayseri, Turkey, BCR-701 was reported as a method validation tool [21]. Two certified reference materials, CRM 141 R and SRM 2709 SJS, were also used to develop method for the urban soils of Algeria [12] while in the industrial soils of Kosovo, CRMs in rock forms (BR, DR-N, and GH) produced by Centre National de la Recherche Scientifique, CRNS (Notre Dame des Pauvres, France), and DTS-1, SDO-1, and AGV-2 produced by the United States Geological Survey, USGS (Denver, Colorado, USA) have been employed to develop suitable analytical methods [16].

## 4. In Depth Analysis of Soil Metal Pollution by Industry Types

As one of the major indices of environmental pollution, trace element concentration data in the soil phase affected by industrial activities complied in this study is listed in Table 3. The comparable data sets for the air and water, although much more limited relative to soil matrix, are also provided in Table 4. In light of differences in the relative abundance of metal pollution data between these different matrices, we conducted a detailed analysis of the impact of industrial activities on environmental metal pollution by focusing mainly on soil media.

Trace metals occur naturally in soils as a result of diverse geological processes such as chemical reaction and erosion of underground geological materials [56]. Beside these natural sources, industrial activities can supply a considerable quantity of metals to soil [57]. A large number of industrial activities produce wastes and contaminants that reach the soil through direct disposal, spills, leaks, atmospheric deposition from air, and other pathways [25]. Hence, enhanced metal levels (e.g., Cu, Zn, Pb, Co, Ni, Cd, As, and others) in soil media have been reported from in and around several industrial sites. As one of the dominant transportation routes of heavy elements, atmospheric emissions have commonly been designated as the main route of metallic accumulation in surface soils via their subsequent deposition, along with other transport routes like waste water discharge [58].

### 4.1. Mining, Smelter, and Metallurgical Industries

Severe metal pollution has been reported from areas surrounding mines and smelters in many countries [33, 59–63]. High levels of metals discharged from mine wastes may cause adverse environmental effects, because they can be dispersed into nearby agricultural soils and stream systems and taken up by food crops [64]. Among the 17 references dealing with metal pollution in mining and smelter sites, 4 elements (Cu, Cd, Pb, and Zn) stood out as the most common choice of target analyte. However, the data for other metals (e.g., Mn, Ni, Cr, Fe, and As), although not as common as the above 4 elements, are also available in many studies (Table 3). For this industry type, noticeably high concentrations of Pb, Zn, Mn, and Cr are frequently detected in the soil samples analysed. The relative ordering of the metal concentrations in these soils, if compared in terms of their median values, can be arranged in the following descending order: Mn > Zn > Pb > Cr > Ni > Cu > As > Fe > Cd (Table 5).

Fairly high concentrations of Mn, Zn, and Cr (e.g., 443, 68.3, and 98.7 mg kg^−1^, resp.) were found near metal industries in Thessaloniki [17]. In general, all elements determined in their study were comparable to the levels normally determined in clay soils worldwide [17]. Enhanced concentrations (mg kg^−1^) of Mn (652), Pb (85), Zn (92), and Cu (47) were also found in soils surrounding the mining and smelting areas in Tharsis, Spain [38]. An investigation covering eight smelters and mining sites in Albania reported exceedingly high concentrations of one or more metals [11]. These authors reported the maximum concentrations (mg kg^−1^) of metals in soil dry matter (DM): Cd (14), Cr (3,865), Cu (1,107), Ni (3,579), Pb (172), and Zn (2,495). If one refers to the report of Kabata-Pendias and Pendias [65], the measurements of Shallari et al. [11] appear to be toxic with the observed levels harmful for plant growth.

Borgna et al. [16] measured 12 trace elements (As, Cd, Co, Cr, Cu, Ni, Pb, Sb, Th, Tl, U, and Zn) in topsoils from the smelter site in the K. Mitrovica area, Kosovo. They reported considerably elevated median values (mg kg^−1^) for Pb, Zn, and Cu of 294, 196, and 37.7, respectively. Borgna et al. [16] also noticed that metal levels caused by mining activities decreased significantly with soil depth. Such pollution activities, therefore, basically affected the upper soil layer between 0 and 50 cm [66]. The analysis of vertical soil profiles generally showed an accumulation of trace metals towards the surface soil due to the outputs of mining, smelter, and metallurgical industries [61, 67, 68]. However, there is also contrasting evidence of vertical metal distribution patterns. For instance, near an iron smelter in Bulgaria, Schulin et al. [15] found that the differences in metal levels between topsoil and subsoil samples were generally small. These authors concluded that the observed vertical profiles of soil metal levels in the study area should primarily be related to geogenic processes. Martley et al. [33] was also unable to find any differences in metal concentrations at soil layers between 0–5 and 5–20 cm, except for Pb and Zn. Similar partitioning results for trace elements were also seen from soils around the Harjavalta smelter, Finland [69], and in Tharsis, Spain [38].

Considering horizontal distributions, metal concentrations in soil usually decrease with increasing distance from the mining or smelter site, generally following an exponential or negative-power decay function. In most cases, metal concentrations in topsoil layers significantly exceeded those of background levels (background levels being considered as those many kilometers from the smelter facilities). For instance, contamination was found to be detectable up to 33 km for a copper smelter or up to 217 km for a zinc smelter in Canada [70]. It was found that As was generally transported over long distances relative to other elements (e.g., Pb, Zn, etc.) [68, 71]. The reason for such enhanced distribution of As may come as a result of its higher volatility and extended atmospheric residence time. Hence, As is less likely to deposit very near the point source from which it was emitted [72]. However, smelter emissions generally depend on a variety of factors including the mass of emitted contaminants, their particle size distributions, stack height, meteorological conditions such as wind speed and direction, and topography [67]. Garmash [73] found that concentrations of zinc, lead, and cadmium were an order of magnitude lower in soil at iron smelter than from a nearby the zinc smelter as a function of the different emissions profile from each plant.

### 4.2. Chemical and Petrochemical Industries

Chemical and petrochemical industries have been identified as large emitters of not only metals but also a wide variety of pollutants (e.g., volatile organic compounds (VOCs), polycyclic aromatic hydrocarbons (PAHs), polychlorinated biphenyls (PCBs), etc.) [25]. As these pollutants are also commonly recognized as markers of environmental pollution, they have also been identified as the cause of some adverse health effects in workers and people living nearby [25]. In Table 3, it is shown that Cd, Pb, Cr, and As are the most abundant trace metals found in soil samples around chemical and petrochemical sites. The release of metals such as Pb, As, or Cr may occur in refining operations and from the burning of residual oils [74]. In contrast, Mn, Ni, Zn, and Cu were determined only at 3, 2, 1, and 1 sites, respectively, from the 7 references examined in this study (Table 3). As shown in Table 5, the mean concentrations (mg kg^−1^) of Pb, Cr, As, and Cd resulting from this industry type were 25.2, 16.6, 5.97, and 0.51, respectively.

In a study conducted near a sulphuric acid plant in Bangladesh, very high concentrations (mg kg^−1^) of Zn (126), Mn (277), Ni (88.2) and Cu (63.5) were detected in soil samples [23]. The status of metal pollution in soil near the sulphuric acid production facilities should directly depend on the quantity of waste material discharged onto the land. Furthermore, many trace metals are likely to be deposited by localized acid rain, once emitted to air from the industry in question [23]. There is also a strong possibility of soil acidification as a result of SO_2_ emissions from the acid-producing industries [75]. Low pH values from the sulphuric acid production facility are also likely to help increase solubility and mobility of metals in the soil.

### 4.3. Textile Industries

Textile industries can act as one of the major sources of metal pollution in the environment [41]. There is evidence that significant amounts of trace metals have been released into the surrounding soil from textile industries (Table 3). In one of the previous studies conducted in Bangladesh, mean soil concentrations of Pb, Zn, Mn, Ni, and Cd in the vicinity of textile industries were found to be 56.4, 207, 382, 51.1, 164 mg kg^−1^, respectively [23]. All metals except Ni were detected in 18 soil samples collected near textile industrial facilities with their mean values being (mg kg^−1^) of 191 (Pb), 668 (Mn), 109 (Cu), 586 (Cr), 380 (Fe), and 83.6 (Cd) [41]. In addition, chromium and cadmium in the soils contaminated with textile effluent in Tamil Nadu, India were reported to be in the range of 55.4–180 and 0.2–5.8 mg kg^−1^, respectively [76]. Although relatively high levels of lead are generally seen in soil samples contaminated by textile industries, conversely lead concentrations in textile effluents were below the detection limit. The presence of lead in soil was thus ascribed to its airborne transport and subsequent deposition from automobiles and other industries in the area.

### 4.4. Leather Industries

Solid and liquid wastes emanating from the tanning industry are known to contain various toxic trace metals [68]. In most developing countries, tannery effluents are directly discharged to nearby land where they adversely affect the quality of both soil and ground water [23]. According to our evaluation, Cr showed the highest values followed by Zn, amongst the trace metals reported in this category (Table 5). This finding can be ascribed to the fact that chromium salts are the most widely used tanning substances [32, 77]. Only a fraction of the chromium salts are actually consumed during leather processing, and most of the salt is discharged as liquid effluent [30]. The mean concentrations (mg kg^−1^) of trace metals were determined to be Cr (744), Zn (0.97), Cu (0.04), Fe (37.7), and As (0.04) in soil samples in the vicinity of leather industries in India [41]. On the other hand, higher iron and copper levels were seen (4837–6311 and 7.20–20.5 mg kg^−1^, resp.) in soils affected by tannery effluent in Mexico [78]. Cr was also reported to range from 155 to 568 mg kg^−1^ in tannery waste contaminated soil in the Vellore district of Tamil Nadu, India [5].

### 4.5. Non-Metallic Mineral Industries

Nonmetallic mineral industries such as cement, ceramic, and battery manufacturing facilities can act as a major source of trace metal pollution in soil. In a study made in Jordan, it was suggested that cement factory emissions might represent the most important pollution source in the area investigated [29]. High Pb, Zn, Mn, and Ni concentrations (e.g., 55, 45, 204, and 39 mg kg^−1^, resp.) were recorded in the soil samples close to the cement plant [29]. This may reflect the fact that the process and production of cement industry require a substantial amount of energy (supplied by burning fossil fuel) and traffic activity in and around the plant [79]. Kashem and Singh [23] found Ni and Zn in excess of tolerable levels, set as 50 mg kg^−1^and 290 mg kg^−1^, respectively, in the soil samples of ceramic industry sites in Bangladesh. High levels (mg kg^−1^) of Pb (268) and Zn (169) were also found near battery manufacturing facilities, which are suspected to pollute the soil in the industrial area of Baoji city, China [28]. It was noted that Pb is the main primary raw material used for battery production, while the geochemical behavior of Pb, Zn, and Mn is known to be very similar in most natural processes [80].

## 5. Evaluation of Metal Pollution between Soils Affected by Different Industry Types

**Figure 2:**
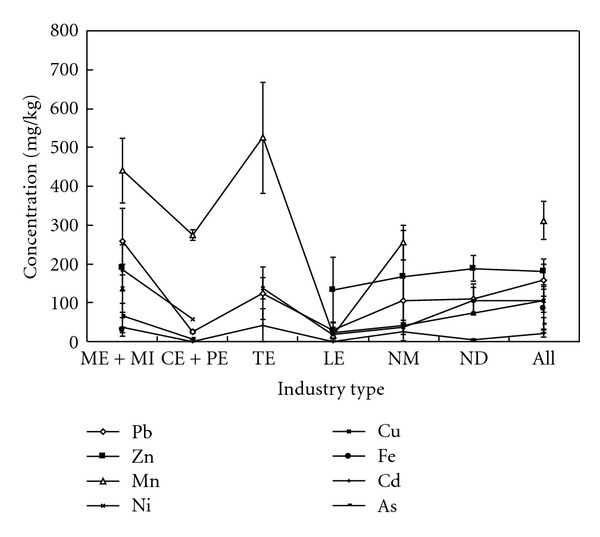
Comparison of soil metal levels affected by different industry types.

To learn more about the relative dominance of a given metal between different industry types, we evaluated our soil metal data by various classification criteria (Table 5). Considering the metal concentration levels determined for different industry types, the overall mean values for different metals can be arranged in the following descending manner: Mn > Zn > Cr > Pb > Ni > Cu > Fe > As > Cd (Table 5). Across the different industry types, the highest average metal concentrations (6 out of 9 studies) were observed from smelter and metal industries. However, the highest mean Mn and Cd concentrations were found from the textile industry, while the highest Cr from leather industry studies (Figure 2). In a study comparing textile and leather industries, Deepali and Gangwar [41] found chromium content to be 23.6% lower in soil contaminated by textile effluent than that contaminated by tannery effluent. Textile industry effluent showed an excess concentration when compared to that from the tannery industry of 87.8% (Fe), 99.9% (Mn), and 99.9% (Cd), while soils affected by tannery industry were characterized by excess Cr. In another study comparing textile and tannery industries in Bangladesh, higher concentrations of Mn, Ni, and Cu were found in soil samples due to textile activity [23]. On the other hand, Pb and Zn showed the opposite trend, that is, higher values from tannery than textile industry samples.

The rapid growth in industrial activities has increased the pressure on environmental sustainability. For a healthy and balanced environment, several regulations have been set out by different governments recognizing the need for improved environmental management. In case of soil, the regulations are set on total metals in the soil, and as such it is important to consider bioavailability of the metals in relation to soil physiochemical properties (such as organic matter content, clay content, cation exchange capacity, etc.). The regulations for the maximum allowable soil metal levels established in different countries are presented in Table 6. However, there are large variations in regulatory metal limits in different countries. In this discussion, we will consider mainly regulatory values which are common to a large number of countries. If our database of soil pollution (Table 3) is examined in this respect, Pb is found to exceed the maximum allowable level (e.g., 100 mg kg^−1^ in Table 6) on many occasions as a result of the metal and mining industries (8 out of 17 studies) and on a single occasion from textile, ceramic, and battery manufacturing industries. Much more emphasis has been placed on lead contamination in soils in recent years, as it exerts very toxic effects on humans and animals. Lead enters human or animal metabolism either via the food chain or by intake of soil dust [93]. In addition to the sources mentioned above, battery production and scrap battery recovery facilities, thermal power plants, and iron-steel industries are commonly found to be major industrial sources of Pb [94]. The concentration of Zn measured from a (Pb and Zn) smelter in China, a metal industry facility in Kosovo, and an industrial complex in India averaged as 907, 560, and 313 mg kg^−1^, respectively (Table 3). Hence, these observed Zn levels also exceeded commonly allowable concentration levels (e.g., 300 mg kg^−1^ in Table 6). The mean concentration of Ni was much higher than its allowable limit (e.g., 50 mg kg^−1^) close to a number of industries such as mining and metal, chemical, textile, ceramic, and industrial complexes. In the case of Cu, concentrations above the regulatory value (e.g., 100 mg kg^−1^) were mainly seen from textile and metal industries. In textile processing, a number of heavy metals (especially Cu, Cr, Zn, etc.) are used in dying and printing processes [95]. As such, the mean concentrations of Cr and Cd in metal, textile, leather, and industrial complexes exceeded the guideline values (i.e., 150 and 3 mg kg^−1^, resp.). The contents of Cr and Cd in topsoil were reported to increase due to the release of various industrial wastes such as tannery wastes, electroplating sludges, leather manufacturing wastes, and so forth [94]. In contrast, exceedance of the regulatory guideline level for As (i.e., 20 mg kg^−1^) was found only in the case of mining and metal industries.

## 6. Assessment of Geoaccumulation (*I*_geo_) Index

In order to learn more about the level of metal pollution in soil and around industrialized areas, we employed a common approach to estimate the enrichment of metal concentrations in soil relative to background concentration, by computing the geoaccumulation index (*I*_geo_) [96, 97]. This method allows assessment of the extent of metal pollution into seven classes based on the increasing numerical value of the index as follows: (1) *I*_geo_ ≤ 0: practically uncontaminated, (2) 0 < *I*_geo_ < 1: uncontaminated to moderately contaminated, (3) 1 < *I*_geo_ < 2: moderately contaminated, (4) 2 < *I*_geo_ < 3: moderately to heavily contaminated (5) 3 < *I*_geo_ < 4: heavily contaminated (6) 4 < *I*_geo_ < 5: heavily to extremely contaminated, and (7) 5 ≤ *I*_geo_: extremely contaminated [96]. This index can be derived by the following equation:

(1) $$I_{\text{geo}}=\log_{2}\left(\frac{Cn}{1.5Bn}\right),$$

where *Cn* is the measured concentration of the trace element in the soil samples and *Bn* is the geochemical background value in the earth's crust. The constant 1.5 allows us to account for the natural fluctuations in the content of a given substance in the environment and the possible influence of nonlocalized anthropogenic sources. Results for calculation of the geo-accumulation index using our survey soil data are summarized in Table 7. If we calculate the average *I*_geo_ values of the trace metals in our soil database, they can be arranged in ascending order: Zn (0.81), Ni (1.26), Cu (1.28), As (1.49), Cr (1.60), Pb (2.06), and Cd (3.60). According to this geo-accumulation index calculation, it can be seen that the extent of soil pollution is the least significant for such metals as Mn and Fe, despite the influence of industrial activities. This indicates that these two metals are commonly derived from natural (geogenic) processes. It is also found that Zn, Ni, and Cu remain below class 3 level, while the highest *I*_geo_ were recorded for Cr, As, Pb, and Cd such as 3.57, 3.84, 5.02, and 9.33, respectively (Table 7). Among the metals, higher *I*_geo_ values were found for Pb and Cd that are mainly in association with mining and metal industries. The relative ordering of *I*_geo_ values within our study is comparable to those reported in a number of previous studies. In one of the previous studies in the Gebze industrial area, Turkey, the highest *I*_geo_ values for metals were found as 10.2 (Cd), 8.38 (Pb), 6.64 (Zn), 4.77 (As), 3.63 (Cu), 3.52 (Mn), and 3.49 (Cr) [94]. Around a cement factory in Ghana, the *I*_geo_ of Ca, Cu, Mn were found 1.21, 1.36, and 2.96, respectively [98].

## 7. Conclusions

To learn more about the effect of industrial activities on environmental pollution, we performed a comprehensive survey of metal pollution in different environmental media. Although database which our survey drew upon is limited in terms of number of studies and the range of industry types, it is representative of the diversity and type of studies in the literature and hence the results of our analysis still provide valuable insights into metal pollution in the soil environment. The data obtained in this survey demonstrate that the metal concentrations in soil generally reflect the influence of various local industrial activities which include metal and mining, chemical and petrochemical, textile, leather, cement, and ceramic industries. Observations of generally enhanced metal levels in soils around various industrial facilities are explainable by unregulated, untreated solid and fluid wastes released by the industries to the nearby land. Among the 9 reference trace metals examined, it is seen that Cu, Cd, Pb, and Zn were monitored the most frequently. Evaluation of soil metal data indicates that their maximum values occur in relation to particular industry types, that is, Pb, Zn, Ni, Cu, Fe, and As in smelter and metal industries, Mn and Cd in the textile industry, and Cr in leather industry studies. The observed metal levels in many of the mining and metal industry samples frequently exceeded the guidance levels set by the environmental legislation in the relevant country. The status of soil pollution in this study, if assessed according to *I*_geo_ values, was classified as moderately-to-extremely contaminated. Most of the samples exhibited the strongest contamination in Pb and Cd. However, the samples were not greatly polluted with respect to Mn and Fe. Considering that there are no regulatory guidelines regarding soil pollution in many developing countries, more efforts should be made to characterize the soil pollution in relation to various industrial activities. This may also help us set proper soil regulation guidelines for sustaining a healthy and balanced environment and protecting human health.

## Figures and Tables

**Table 1:** Classification of industry types for the analysis of soil metal pollution: the International Standard Industrial Classification (ISIC) codes.

Order	Section	Division	Short mane	Groups	Industry Code^a^
1	Manufacturing	Beverages	BE	Beverages	110
2		Textiles	TE	Spinning, weaving, and finishing of textiles	131
3				Other textiles	139
4		Leather and related products	LE	Tanning and dressing of leather, luggage, handbags, saddlery and harness, dressing, and dyeing	151
5		Paper and paper products	PA	Paper and paper products	170
6		Coke and refined petroleum products		Refined petroleum products	192
7		Chemicals and chemical products	CE	Chemicals, fertilizers and nitrogen compounds, plastics, and synthetic rubber in primary forms	201
8		Pharmaceutical products and pharmaceutical preparations	PH	Pharmaceuticals, medicinal chemical, and botanical products	210
9		Nonmetallic mineral products	NM	Glass and glass products	231
10				Nonmetallic mineral products	239
11		Metals	ME	Iron and steel industry	241
12				Precious and other nonferrous metals industry	242
13		Machinery and equipment	MA	General-purpose machinery	281
14		Electrical equipment	BA	Batteries and accumulators	272
15		Transport equipment	TR	Building of ships and boats	301
16		Furniture	FU	Furniture	310

17	Mining and quarrying	Extraction of crude petroleum and natural gas	PE	Extraction of crude petroleum	061
18		Mining of metal ores	MI	Mining of iron ores	071
19				Mining of non-ferrous metal ores	072

^a^Source: ISIC Rev. 4 structure (2008, United Nations Statistics Division) [10].

**Table 2:** Sampling and analytical procedures of metals in soil matrix reported between different studies.

Order	Study area	No. of samples	Sample collection method			Method Instrument		Reference
			Period	Soil depth (cm)	Grid		Reference materials used	
1	Albania	21	Jul ‘95	0–15	—	ICP-AES	CRMs-BCR 142R	[11]
2	Algeria	119	Jan-Feb ‘06	—	1 × 1 km	FAAS^a^, GFS^b^	CRM 141 R, SRM 2709 SJS	[12]
3	Australia	25	Dec ‘00–Feb ‘01	0–5 and 5–20	—	ICP-MS	CRMs-AGAL 10, AGAL 11	[13]
4	Belgium	27	‘93–‘98	0–20	10 m interval	ICP-MS, ICP-AES	CRMs-SRM2710, GBW07411,	[14]
							GBW07311 & SRM1633	
5	Bulgaria	14	Jun ‘04	—	—	XRF	—	[15]
6	Kosovo	82	Jun–Nov ‘02	—	1×1 km	XRF, ICP-MS	CRMs-BR, DR-N, GH, DTS-1,	[16]
							SDO-1, AGV-2, NIST 2709, 2710	
7	Greece	30	‘93–‘94	—	—	INAA^c^ & AAS	IAEA SOIL-7, Pepperbush SRM	[17]
8	Italy	280	—	0–5	—	ICP-MS	—	[18]
9	Jordan	3	Sept ‘03	0–20 and 20–40	—	AAS	—	[19]
10	Peru	6	—	0–10	—	ICP-ES	—	[20]
11	Slovenia	40	—	0–25	—	FAAS	WEPAL 2004.3.3	[21]
12	UK	70	—	0–15	1 km interval	FAAS	—	[22]
13	Bangladesh	53	Nov ‘95	0–5 and 5–15	—	AAS	—	[23]
14	India	30	‘02–‘04	—	—	XRF	SO-1, SO-2, SO-3, SO-4, NGRI-D,	[24]
							NGRI-U	
15	Spain	24	Winter ‘07	0–5	—	ICP-MS & AAS-GF	CRM 052	[25]
16	Spain	27	Winter ‘05	0–3	—	ICP-MS	Lobster hepatopancreas	[26]
17	Turkey	29	Sep ‘03	—	—	FAAS	SRM - BCR-701	[27]
18	China	50	—	0–10	20 m × 20 m	XRF	GSS, GRS, GSD, SO-1, NIST-2709,	[28]
							NIST-2710, NIST-2711	
20	Jordan	31	—	0–10 and 10–20	—	AAS	NBS SRMs	[29]
21	Pakistan	38	Oct ‘03–Dec ‘03	1–5	—	AAS	SRM-SR-96	[30]
22	Serbia	59	Jun ‘99–Mar ‘00	0–5	—	FAAS	—	[31]
23	Damascus	51	Summer ‘98	0–20	—	FAAS	—	[32]

^a^FAAS—Flame atomic absorption spectrometer.

^b^GFS—Graphite furnace spectrometry.

^c^Instrumental Neutron Activation Analysis with gamma-ray spectroscopy.

**Table 3:** Summary of trace element concentrations in soils affected by diverse industrial activities.

Order	Industry type	Industry group short name^a^	ISIC industry code^a^	Location		Trace element concentration (mg kg^−1^)									Reference
				City	Country	Pb	Zn	Mn	Ni	Cn	Cr	Fe	Cd	As	
1	Iron smelter		241	Kremikovtzi	Bulgaria	—	—	—	111	61.8	—	—	2.10	—	[15]
2	Pb and Zn smelter		242	Zhuzhou	China	472	907	—	—	77.0	—	—	11.9	38.0	[28]
3	Zn Smelter		242	Celje	Slovenia	865	230	—	49.0	708	—	—	289	37.0	[21]
4	Smelter		241, 242	Wales	UK	136	183	—	—	92.0	—	—	4.9	—	[22]
5	Smelter		241, 242	Reppel-Bocholt	Belgium	899	137	246	94.0	801	—	—	12.0	—	[14]
6	Smelter		241, 242	Kosovska Mitrovica	Kosovo	973	560	—	214	56.2	418	—	7.2	87.1	[16]
7	Metal industry		241, 242	Potenza	Italy	87.7	62.8	—	—	26.1	—	—	1.31	18.3	[18]
8	Metal industry		241, 242	Annaba	Algeria	53.1	67.5	355	—	39.0	30.9	—	0.44	—	[12]
9	Metal industry	ME + MI	241, 242	Karak ind. Estate (S)	Jordan	15.7	4.9	—	3.70	1.60	—	23.4	—	—	[29]
10	Copper smelter, steel industry		241, 242	Port Kembla	Australia	20	42.0	—	—	49.0	12.0	—	—	3.20	[33]
11	Metal recycling, steel production		241, 242	Murcia	Spain	18.3	21.8	229	17.7	7.40	19.4	—	0.20	—	[37]
12	Ferrous and non-ferrous metal smelters, iron and steel manufacturing, metal recovery, manganese ore treatment, scrap, metal incineration		241, 242	Thessaloniki	Greece	24.2	68.3	443	—	26.8	98.7	37.1	0.22	36.1	[17]
13	Copper mines		072	Canchaque	Peru	156	235	714	801	—	—	—	183	279	[20]
14	Smelters, mining		071, 072, 241, 242	Elbasan	Albania	80	61.0	—	447	14.0	491	—	3.00	—	[11]
15	Smelters, mining		071, 072, 241, 242	Rubik	Albania	135	250	—	66.0	111	256	—	9.00	—	[11]
16	Smelters, mining		071, 072, 241, 242	Munelle	Albania	103	111	—	54.0	73.0	91.0	—	2.00	—	[11]
17	Smelters, mining		071, 072, 241, 242	Tharsis	Spain	85.0	92.0	654	—	47.0	—	—	—	37.0	[38]

18	Petrochemical		061	Tarragona	Spain	29.5	—	297	—	—	20.4	—	0.19	4.71	[25]
19	Petrochemical		061	Almowra	Spain	—	—	—	27.6	—	—	—	2.13	—	[39]
20	Petrochemical		061	Tarragona	Spain	36.3	—	—	—	—	13.8	—	0.21	5.50	[26]
21	Petrochemical	CE + PE	061	Catalonia	Spain	37.8	—	—	—	—	16.5	—	0.21	6.51	[40]
22	Chemical		201	Tarragona	Spain	24.5	—	248	—	—	14.8	—	0.17	6.87	[25]
23	Chemical		201	Valencia	Spain	22.2	—	—	—	—	17.5	—	0.16	6.24	[40]
24	Sulphuric acid-producing industry		201	Dhaka	Bangladesh	1.03	126	277	88.2	63.5	—	—	0.53	—	[23]

31	Textile industries	TE	131,139	Dhaka	Bangladesh	56.4	207	382	51.1	164	—	—	0.48	—	[23]
32	Textile industries		131,39	Haridwar	India	191	—	668	—	109	568	308	83.6	—	[41]

27	Tannery industries		151	Dhaka	Bangladesh	68.1	290	39.7	5.20	17.6	—	—	1.26	—	[23]
28	Tannery industries	LE	151	Haridwar	India	—	—	097	—	0.04	744	37.7	0.04	—	[41]
29	Tannery industries		151	Peshawar	Pakistan	4.66	2.38	5.29	—	—	29.9	18.0	0.60	—	[30]
30	Tannery industries		151	Damascus	Syria	17.0	103	—	39.0	34.0	57.0	—	—	—	[32]
33	Cement factory		239	Qadissiya	Jordan	55.0	45.0	204	39.0	2.90	22.2	—	5.00	—	[29]
25	Ceramic industries	NM	239	Dhaka	Bangladesh	28.6	287	217	50.1	38.4	—	—	0.33	—	[23]
26	Ceramic industries		239	Castellón	Spain	229	—	345	36.5	66.0	29.7	—	72.0	—	[42]

34	Battery manufacturing	BA	272	Baoji	China	268	169	—	39.3	62.3	100	—	—	—	[28]

35	Textile, plastic, furniture, industries		131, 201, 310	Thane-Belapur	India	—	191	—	184	105	521	—	—	—	[43]
36	Chemicals, dyes, textile, paint industries		131, 201, 202	Rajasthan	India	293	—	—	136	298	240	—	—	—	[43]
37	Paint, battery, textile, milling and chemical industries		131, 201, 272	Lagos	Nigeria	—	—	—	24.7	106	—	—	13.4	—	[44]
38	Ceramic, paper products, petrochemical industries		061, 170, 239	Belgrade	Serbia	37.7	103	—	66.6	40.0	—	—	—	—	[31]
39	Chemicals, pharmaceutical, batteries	ND	201, 210, 272	Hyderabad	India	65.0	313	—	45.0	193	433	—	0.70	33.0	[24]
40	Chemicals, furniture, printing, leather, textile		131, 151, 201, 310	Kayseri	Turkey	74.8	112	—	44.9	36.9	29.0	—	2.53	—	[27]
41	Brewery, steel/metal works, paints, pharmaceuticals, packaging, food processing, textiles and plastic products industries Tannery, textile, fertilizer,		110, 131, 139, 201, 241	Lagos	Nigeria	143	247	283	17.0	25.6	26.6	—	2.90	—	[45]
42	rerolling and casting, chemicals paints plastics		131, 139, 151, 201, 241	Jajmau, Unnao	India	38.3	159.9	—	—	42.9	265	—	—	—	[46]

^a^Refer to Table 2.

^b^ND—not defined due to the complexity of industry types.

**Table:** (a) Air phase

Order	Industry type	Industry group Short name^a^	ISIC industry code^a^	Location		Trace element concentrations (ng m^−3^)									Reference
				City	Country	Pb	Zn	Mn	Ni	Cu	Cr	Fe	Cd	As	
1	Petrochemical	CE + PE	061	Tarragona	Spain	1.22	—	4.70	—	—	2.19	—	0.05	0.12	[25]
2	Petrochemical		061	Rio de Janeiro	Brazil	8.90	25.1	1.50	—	12.1	—	15.9	0.30	—	[47]
3	Chemical and petrochemical		061, 201	Rio de Janeiro	Brazil	15.9	2124	16.0	2.10	22.0	2.40	775	0.40	—	[48]
4	Chemical		201	Tarragona	Spain	3.20	—	8.50	—	—	2.19	—	0.23	0.69	[25]

5	Machinery, metallurgical, petrochemistry, pulp and paper, textile		131, 170, 241, 242, 281,	Sihwa	Rrpublic of Korea	321	657	161	—	—	69.6	1203	7.10	7.70	[49]
6	Textile, metallurgical, paper		131, 241, 242, 281,	Banwol	Rrpublic of Korea	346	1272	86.3	—	—	21.0	1218	6.30	6.30	[49]
7	Metal recycling, steel production		241, 242	Pohang	Rrpublic of Korea	93.8	389	245	—	—	23.9	5423	1.95	—	[50]
8	Ceramic, paper products, petrochemical	ND^b^	061, 170, 239	Onda	Spain	164	203	7.00	—	—	3.00	432	—	—	[40]
9	Oil refineries, cement, shipyards, steel		192, 239, 241, 301	Elefsina	Greece	71.1	—	21.1	—	—	—	—	14.5	3.70	[51]
10	Oil refineries, steel mill Ferrous and non-ferrous metal		192, 241	Capana	Argentina	70.6	36.9	21.2	—	—	2.83	552	0.27	2.76	[44]
11	manufacturing, fertilizer production, manganese ore treatment, scrap, metal incineration, oil refining		192, 201, 241, 242	Thessaloniki	Greece	140	830	84.0	—	59.0	7.50	—	2.70	3.60	[17]

^a^Refer to Table 2.

^b^NO—not defined due to the complexity of industry types.

**Table:** (b) Water phase

Phase	Industry type	Industry group short name^a^	ISIC industry	Location		Trace element concentration (mg L^−1^)									Reference
			code^a^	City	Country	Pb	Zn	Mn	Ni	Cu	Cr	Fe	Cd	As	
1	Brewery		110	Ibadan	Nigeria	—	0.33	0.04	—	0.60	0.01	0.18	—	—	[52]
2	Brewery	BE	110	Benin	Nigeria	—	0.17	0.03	—	0.46	0.01	0.38	—	—	[52]
3	Brewery		110	Lagos	Nigeria	—	0.22	—	—	0.54	0.02	0.15	—	—	[52]
4	Brewery		110	Ibadan	Nigeria	0.19	2.40	—	—	0.36	0.21	—	0.11	—	[53]

5	Tannery	LE	151	Peshawar	Pakistan	0.08	0.13	0.087	—	—	0.09	0.14	0.014	—	[30]
6	Tannery		151	Haridwar	India	—	—	0.02	—	—	0.93	0.84	0.007	—	[41]

7	Textile	TE	131,139	Kaduna	Nigeria	—	0.31	1.18	—	1.16	—	2.14	—	—	[54]
8	Textile		131,139	Haridwar	India	—	—	0.7	—	0.05	—	2.95	—	—	[41]

^a^Refer to Table 1.

**Table 5:** Statistical summary of trace element concentration (in mg kg^−1^) in soils affected by industrial activities.

Industry group short name	Statistical parameter	Trace elements								
		Pb	Zn	Mn	Ni	Cu	Cr	Fe	Cd	As
ME + MI	Mean	258	190	440	186	137	177	30.3	37.6	67.0
	Median	95	102	399	80.0	52.6	94.9	30.3	3.95	37.0
	SD	343	234	205	252	244	189	9.69	86.7	89.0
	Min	15.7	4.90	229	3.70	1.60	12.0	23.4	0.20	3.20
	Max	973	907	714	801	801	491	37.1	289	279
	*N*	16	16	6	10	16	8	2	14	8

CE + PE	Mean	25.2	126	274	57.9	63.5	16.6	—	0.51	5.97
	Median	25.2	—	274	57.9	—	16.6	—	0.51	5.97
	SD	13.4	—	24.6	42.9	—	2.57	—	0.72	0.86
	Min	1.03	—	248	27.6	—	13.8	—	0.16	4.71
	Max	37.8	—	297	88.2	—	20.4	—	2.13	6.87
	*N*	6	1	3	2	1	5	0	7	5

TE	Mean	124	207	525	51.1	136	568	308	42.0	—
	Median	124	—	525	—	136	—	—	42.0	—
	SD	95.2	—	202	—	38.7	—	—	58.8	—
	Min	56.4	—	382	—	109	—	—	0.48	—
	Max	191	—	668	—	164	—	—	83.6	—
	*N*	2	1	2	1	2	1	1	2	0

LE	Mean	29.9	132	15.3	22.1	17.2	277	27.9	0.63	—
	Median	17.0	103	5.29	22.1	17.6	57.0	27.9	0.60	—
	SD	33.6	146	21.2	23.9	17.0	405	13.9	0.61	—
	Min	4.66	2.38	0.97	5.20	0.04	29.9	18.0	0.04	—
	Max	68.1	290	39.7	39.0	34.0	744	37.7	1.26	—
	*N*	3	3	3	2	3	3	2	3	0

NM	Mean	104	166	255	41.9	35.8	26.0	—	25.8	—
	Median	55.0	166	217	39.0	38.4	26.0	—	5.00	—
	SD	109	171	77.9	7.24	31.6	5.30	—	40.1	—
	Min	28.6	45.0	204	36.5	2.90	22.2	—	0.33	—
	Max	229	287	345	50.1	66.0	29.7	—	72.0	—
	*N*	3	2	3	3	3	2	0	3	0

BA	Value	268	169	—	39.3	62.3	100	—	—	—

ND	Mean	109	188	283	74.0	106	252	—	4.89	—
	Median	69.9	176	—	45.0	74	253	—	2.72	—
	SD	98.2	81.2	—	62.3	95.6	203	—	5.76	—
	Min	37.7	103	—	17.0	25.6	26.6	—	0.70	—
	Max	293	313	—	184	298	521	—	13.4	—
	*N*	6	6	1	7	8	6	0	4	0

All	Mean	158	180	312	106	106	176	84.8	21.6	42.8
	Median	65.0	131	280	49.6	52.6	44.0	37.1	2.00	25.7
	SD	247	180	208	168	176	217	125	59.8	71.8
	Min	1.03	2.38	0.97	3.70	0.04	12.0	18.0	0.04	3.20
	Max	973	907	714	801	801	744	308	289	279
	*N*	37	30	18	26	34	26	5	33	14

**Table 6:** Regulatory levels for soil metals established between different countries.

Order	Country	Concentration (mg kg^−1^)							Reference
		Pb	Zn	Ni	Cu	Cr	Cd	As	
1	Australia	—	200	60	60	50	—	20	[81]
2	Japan	—	—	—	125	—	1	15	[82]
3	Taiwan	100	300	—	150	200	4	20	[83]
4	Turkey	150	500	—	100	—	5	—	[84]
5	EU	300	300	75	140	150	3	—	[85]
6	Netherlands	150	500	100	100	250	5	—	[86]
7	Spain	300	450	112	210	150	3	—	[87]
8	Germany	100	300	50	100	100	3	20	[88]
9	France	100	300	50	100	150	2	20	[89]
10	UK	550	280	35	140	600	4	10	[90]
11	USA	150	300	31	45	212	2	5.6	[91]
12	Canada	500	500	100	100	250	5	—	[92]

**Table 7:** Comparison of the geoaccumulation index calculated for the soil bound metals affected by industrial activities.

Order	Industry type	Industry group short name^a^	Country	Index of geoaccumulation (*I *_geo_)								
				Pb	Zn	Mn	Ni	Cu	Cr	Fe	Cd	As
1	Iron smelter		Bulgaria	—^a^	—	—	0.12	—	—	—	2.22	—
2	Pb and Zn smelter		China	3.98	2.67	—	—	0.19	—	—	4.72	0.96
3	Zn Smelter		Slovenia	4.85	0.69	—	—	3.39	—	—	9.33	0.92
4	Smelter		UK	2.18	0.36	—	—	0.45	—	—	3.44	—
5	Smelter		Belgium	4.91	—	—	—	3.57	—	—	4.74	—
6	Smelter		Kosovo	5.02	—	—	1.07	—	1.63	—	4.00	2.16
7	Metal industry		Italy	1.55	—	—	—	—	—	—	1.54	—
8	Metal industry		Algeria	0.82	—	—	—	—	—	—	—	—
9	Metal industry	ME + MI	Jordan	—	—	—	—	—	—	—	—	—
10	Copper smelter, steel industry		Australia	—	—	—	—	—	—	—	—	—
11	Metal recycling, steel production Ferrous and non-ferrous metal smelters, iron and		Spain	—	—	—	—	—	—	—	—	—
12	steel manufacturing, metal recovery, manganese ore treatment, scrap, metal incineration		Greece	—	—	—	—	—	—	—	—	0.89
13	Copper mines		Peru	2.38	0.72	—	2.97	—	—	—	8.67	3.84
14	Smelters, mining		Albania	1.42	—	—	2.13	—	1.86	—	2.74	—
15	Smelters, mining		Albania	2.17	0.81	—	—	0.71	0.92	—	4.32	—
16	Smelters, mining		Albania	1.78	—	—	—	0.11	—	—	2.15	—
17	Smelters, mining		Spain	1.50	—	—	—	—	—	—	—	0.92

18	Petrochemical		Spain	—	—	—	—	—	—	—	—	—
19	Petrochemical		Spain	—	—	—	—	—	—	—	—	—
20	Petrochemical		Spain	0.28	—	—	—	—	—	—	—	—
21	Petrochemical	CE + PE	Spain	0.33	—	—	—	—	—	—	—	—
22	Chemical		Spain	—	—	—	—	—	—	—	—	—
23	Chemical		Spain	—	—	—	—	—	—	—	—	—
24	Sulphuric acid producing industry		Bangladesh	—	—	—	—	—	—	—	0.24	—

31	Textile industries	TE	Bangladesh	0.91	0.54	—	—	1.28	—	—	0.09	—
32	Textile industries		India	2.67	—	—	—	0.69	2.07	—	7.54	—

27	Tannery industries		Bangladesh	1.18	1.03	—	—	—	—	—	1.49	—
28	Tannery industries	LE	India	—	—	—	—	—	2.46	—	—	—
29	Tannery industries		Pakistan	—	—	—	—	—	—	—	0.42	—
30	Tannery industries		Syria	—	—	—	—	—	—	—	—	—

33	Cement factory		Jordan	0.87	—	—	—	—	—	—	3.47	—
25	Ceramic industries	NM	Bangladesh	—	1.01	—	—	—	—	—	—	—
26	Ceramic industries		Spain	2.93	—	—	—	—	—	—	7.32	—

34	Battery manufacturing	BA	China	3.16	0.25	—	—	—	—	—	—	—

35	Textile, plastic, furniture, industries		India	—	0.42	—	0.85	0.63	1.95	—	—	—
36	Chemicals, dyes, textile, paint industries Paint, battery, textile,		India	3.29	—	—	0.42	2.14	0.83	—	—	—
37	milling and chemical industries		Nigeria	—	—	—	—	0.65	—	—	4.90	—
38	Ceramic, paper products, petrochemical industries Chemicals, pharmaceutical, batteries		Serbia	0.33	—	—	—	—	—	—	—	—
39	plastic products	ND	India	1.12	1.14	—	—	1.52	1.68	—	0.64	0.76
40	Chemicals, furniture, printing, leather, textile Brewery, steel/metal works, paints,		Turkey	1.32	—	—	—	—	—	—	2.49	—
41	pharmaceuticals, packaging, food processing, textiles and plastic products industries Tannery, textile,		Nigeria	2.25	0.80	—	—	—	—	—	2.69	—
42	fertilizer, rerolling and casting, chemicals, paints, plastics		India	0.35	0.17	—	—	—	0.97	—	—	—

^a^No numeric values are shown for the cases with negative values.
